# The Importance of Quality Assurance in Trace Analysis

**DOI:** 10.6028/jres.093.026

**Published:** 1988-06-01

**Authors:** John K. Taylor

**Affiliations:** National Bureau of Standards, Gaithersburg, MD 20899

## 1. Introduction

The quality of data must be known if it is to be used in any logical sense in any decision process. Data must be both technically sound and defensible. This simple statement seems axiomatic, yet its impact only recently has been widely recognized. And even today, much of the data generated for environmental, health, and other vital purposes is of questionable quality.

Data quality has impact on the attainable accuracy of every measurement. In trace analysis, which is often pushing the lower limits of measurement, it influences the decision of whether the measured value has any significance, whatsoever.

**Figure 1:**
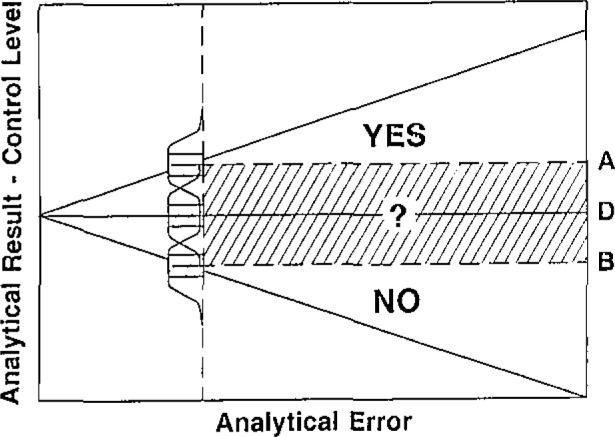
Effect of data quality on analytical decision.

The effect of data quality on any analytical decision is illustrated in figure 1. The total uncertainty is indicated by the bounds for bias (dotted areas) and the random component (curve enclosed areas). A value just at the decision level, D, has some probability of being either larger or smaller than that *measured*. Even a value of A, well above D, has a probability of being smaller, changing the decision from YES to NO, and conversely for a value B. The indecision zone is clearly a function of imprecision and bias, both of which must be known and estimated.

**Figure 2:**
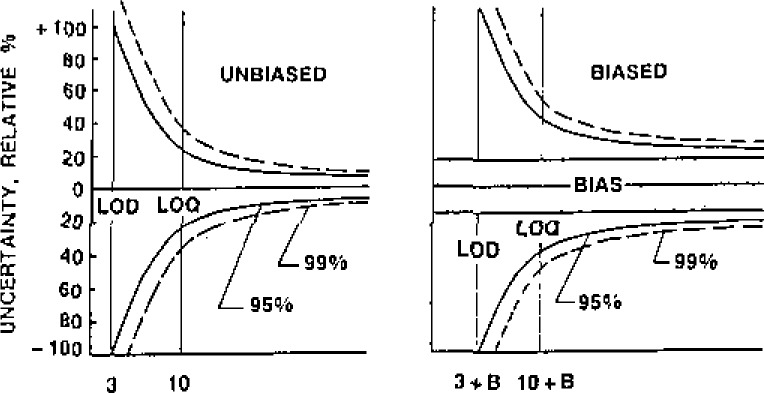
Effect of data quality on decision of selection.

A similar situation exists for the decision of detection, shown in figure 2. Detection consists in whether a measured value is larger than its uncertainty, and data are only quantitatively useful if their relative uncertainty is reasonably smaller than the measured values. The limit of detection (LOD) and the limit of quantitation (LOQ) depend on the magnitude of the standard deviation and the bounds for bias. The limits in figure 2 correspond to those recommended by the American Chemical Society, Committee on Environmental Measurement [1].

It should be clear that data uncertainty must be known and the measurement system must be stable if data are to be used with confidence. Their quality must be assured by the producer and to the user(s). Until a measurement system has achieved statistical control, it cannot be considered in any logical sense as measuring anything at all [2].

## 2. What is Quality Assurance?

Quality assurance consists of all activities undertaken to produce data of evaluated quality [3]. It consists of two separate but related activities: Quality Control—What is done to obtain data of acceptable quality, and Quality Assessment—What is done to evaluate the quality of the data produced.

## 3. Quality Control

All sources of variability of the measurement process must be stabilized and optimized, consistent with the end use of the data. Bias control must be implemented as well. All of these become extremely critical in trace analysis. The major sources of imprecision and bias are shown in table 1. The impacts indicated are for the average case and will differ in importance according to the situation and the degree of control that is achieved.

Quality control is achieved by careful attention to all factors that affect uncertainty. These may be classified in the general categories shown in table 2 [4]. The emphasis is on doing what is necessary, doing it right, and doing it consistently. The latter is achieved by following optimized protocols. While most of the elements in table 2 are self evident, the acronyms may be less familiar.

GLPs—Good Laboratory Practices: protocols that define how general operations are to be carried out.
GMPs—Good Measurement Practices: protocols that define how technique-specific operations are to be carried out.
SOPs—Standard Operations Procedures: protocols that define how measurement and sampling operations are to be carried out.
PSPs—Protocols for Specific Purposes: protocols that define how an entire measurement program is to be carried out.

## 4. Quality Assessment

The elements of quality assessment are shown in table 3. They are rated according to their importance in evaluating imprecision (P) and bias (B), and also whether they can be provided by a laboratory’s internal (I) resources or whether they are provided externally (E). It is evident that precision evaluation, a prerequisite to bias evaluation, should be relatively easy for a laboratory to accomplish without external assistance. Bias evaluation can be done internally, but is is time consuming and requires external inputs if it is to be objective. Both kinds of evaluation must be consistently monitored, preferably using control charts. The consistent measurement of reference materials is the procedure of choice whenever possible. The importance of audits, both internal and external, to monitor both performance and conformance to quality assurance standards is obvious. And, of course, every concerned analyst and analytical laboratory must critically review its outputs (introspection).

## 5. Conclusion

Quality assurance must be extended to every operation that affects the final measured result if valid conclusions are to be made. That is to say the sample, the sampling operation, blanks, calibration, and measurement.

One must always distinguish between attainable accuracy and the accuracy actually attained. The state-of-the-process is often significantly less than the state-of-the-art, resulting from ill-defined tolerances, inadequate control, poorly designed measurement system, and inappropriate use of methodology. All can become major limitations in trace analysis.

Quality is only achieved by consistent and diligent effort and it is best achieved by developing and following an appropriate QA program. But QA is more than a system. It is a philosophy, and indeed a way of life. As a system, it is doomed to disappointment, if not failure. As a philosophy, there is some hope for success. As a system and a philosophy, that is to say a way of life, the prospects for quality data are excellent [5].

## Figures and Tables

**Table 1:** Sources of uncertainty in trace measurements

Source	Impact	Precision	Bias
Sample	high-to-extensive	m	M
Sub-sampling	high	M	M
Chemical operations	High	M	M
Losses	high	m	M
Contamination	medium-to-high	M	M
Blank	medium-to-high	m	M
Calibration	medium-to-high	m	M
Instrumentation	low-to-medium	m	m

m = minor contributor

M = major contributor

**Table 2:** Elements of quality control^a^

Competent personnel
Suitable facilities
Appropriate methodology
Adequate calibration
GLPs
GMPs
SOPs
PSPs
Inspection
Documentation

a See reference [4] for a detailed discussion of the elements.

**Table 3:** Elements of quality assessment

Element	Impact	Source	
Replicate measurements	P	I	
Reference materials	B		E
Definitive measurements	B	I	E
Independent methods	B	I	
Spikes	B	I	
Surrogates	B	I	
Collaborative testing	B		E
Audits	P/B	I	E
Control charts	P/B	I	
Introspection	P/B	I	
